# Prevalence of familial hypercholesterolemia in patients with confirmed premature coronary artery disease in Ranchi, Jharkhand

**DOI:** 10.1186/s43044-022-00320-7

**Published:** 2022-12-17

**Authors:** Prakash Kumar, Shashi Ranjan Prasad, Anushil Anand, Rajneesh Kumar, Sajalendu Ghosh

**Affiliations:** 1 Department of Cardiology, RIMS Hospital, Ranchi, Jharkhand India; 2 Department of Zoology, Dr. Shyama Prasad Mukherjee University, Ranchi, Jharkhand India

**Keywords:** Acute coronary syndrome, Arcus cornealis, Coronary artery disease, Familial hypercholesterolemia, Xanthomas

## Abstract

**Background:**

Familial hypercholesterolemia (FH) is an under-diagnosed autosomal co-dominant genetic disorder characterized by very high plasma levels of low-density lipoprotein cholesterol (LDL-C), premature coronary artery disease (CAD) with arcus cornealis, and xanthomas. Among patients with CAD, the frequency of FH is significantly higher than that of the general population, but little data are available in India in this regard. This study aimed to assess the prevalence of FH in patients with premature coronary artery disease for the first time in the Jharkhand population.

**Results:**

The study was conducted on 200 premature CAD patients at RIMS hospital, Ranchi, from January 2020 to June 2021 with CAG-confirmed acute coronary syndrome. The study, without taking the aid of genetic profiling of the patients and using the Dutch Lipid Clinic Network Criteria, revealed quite a high (23.5%) prevalence of potential FH in patients with premature CAD apart from the conventional risk factors. Mean LDL-C levels among patients with definite, probable, possible, and no FH were recorded as 250.39, 184.32, 136.11, and 108.09 mg/dl, respectively. Arcus cornealis was seen in 55.31% of patients with potential FH, 90% in definite FH, and 44.40% with probable FH. Patients with potential FH were more likely to be younger (age < 40 years) males, having a history of CAD and a family history of premature CAD as compared to patients without FH.

**Conclusions:**

There was no previous report of large studies on FH or its epidemiology and its natural history from India. The present study is the first one to show a high prevalence of potential FH in premature CAD (about 23.5%). This preliminary study revealed that the prevalence of FH in patients with premature CAD who came to the tertiary care hospital of Ranchi, Jharkhand, was high, apart from the conventional risk factors.

## Background

Familial hypercholesterolemia (FH) is an under-diagnosed autosomal co-dominant genetic disorder characterized by very high plasma levels of low-density lipoprotein cholesterol (LDL-C), xanthomas, arcus cornealis (AC), and premature coronary artery disease (CAD). Tendon xanthomata (TX) are widely regarded as a specific physical sign, but are often absent and may be difficult to assess. FH increases the risk of early CAD by up to 20-fold [1, 2]. It is the most common monogenic disorder leading to premature CAD and cardiac death.

Among patients with CAD or other atherosclerotic diseases, the frequency of FH is significantly higher than that of the general population showing that these patients are at particularly elevated risk of recurrent events [3]. In different ethnic groups across the USA, the prevalence of FH varies [4] and less than 10% of individuals with FH were diagnosed [5, 6]. The estimated prevalence of heterozygous FH (HeFH) is widely variable from 1/200 to 1/500 which contributes to its diagnostic difficulty [7, 8], and homozygous FH (HoFH) is 1/10^6^ in the general population in the west [1]. There are very little data in India about the prevalence of FH. FH is primarily caused by mutations in the gene encoding the low-density lipoprotein receptor (LDLR). Less frequent mutations in the APOB and PCSK9 genes have similar functional consequences [9].

The Dutch Lipid Clinic Network criterion has been used extensively to identify the HeFH phenotype, which is based on LDL-C level, family history of FH, presence of tendon xanthoma, and arcus cornealis. Diagnosis of FH is a key factor in the prevention as this would lead to early recognition of FH in a family member of the index case and in preventing the morbidity and mortality due to CAD by lifestyle modification and judicial usage of hypolipidemic drugs.

The most efficient method of cascade testing for FH is to look for genotyping to relatives of index patients with an identified mutation, but it is in its infancy at the present situation in India. This is better than using family cholesterol testing alone as it is nonspecific [10].

The underdiagnosis of FH in the general population has been recognized as an important issue. For many patients, unaware of the disease, the first clinical manifestation may be acute coronary syndrome (ACS). The number of large-scale studies of FH or its epidemiology and natural history reported from India is close to nil. Although many studies have been conducted in India to study the lipid profile in acute coronary syndrome (ACS), there has been little mention of FH in these publications. There are huge gaps among Asian countries about the knowledge, frequency of occurrence, and care of FH. To fill this gap, it was aimed to assess the prevalence of FH in patients with premature coronary artery disease.

## Methods

This study was conducted on 200 patients of premature CAD, who were admitted to the Ward/CCU of the Department of Cardiology RIMS, Ranchi, Jharkhand, from January 2020 to June 2021 with CAG-confirmed acute coronary syndrome (ACS). ACS is defined as severe chest pain or equivalent ischemic discomfort (dyspnea and epigastric discomfort) and has at least one of three features: (1) It occurs at rest (or with minimal exertion) lasting for more than 10 min. (2) It is of recent onset (i.e., within the prior 2 weeks), and/or (3) it occurs with a crescendo (upsurge) pattern.

### Sample size calculation

Based on clinical experience and study of the literature, the prevalence of the FH in premature CAD was expected to be about 15%. The sample size was calculated with a 5% margin of error and a 5% level of significance. The required minimum sample size was found to be 180 as per the following formula.

Formula used:

$$n = Z^{2}_{\alpha /2} \,pq/d^{2}$$

*p* is the expected prevalence of Familial hypercholesterolemia in patients of premature coronary artery disease and:

$$q = 1 - p$$

*d* is the margin of error.

*Z*_*α*/2_ is the ordinate of standard normal distribution at *α*/2% level of significance.

### Inclusion criteria

Patients of premature CAD with age 45 years or less for males and age less than 55 years for females based on the study by van Loon et al. [11].

### Exclusion criteria

- Patients with advanced liver/kidney disease.
- Pregnant and lactating women.
- Patients with hypothyroidism.
- Patients with cancer

### Study methodology

Patients fulfilling the inclusion criteria were enrolled in the study.

### Data collection

The following data were collected:

- Demographics: name, age, gender, address, etc.
- Clinical history and risk factors for coronary artery diseases.
- A family history of premature coronary artery diseases, high LDL-C, and tendon xanthomas.
- A physical examination of the patient was carried out to look for clinical features of FH such as hypercholesterolemia, tendon xanthomas, arcus cornealis, coronary artery disease, peripheral artery disease, and cerebrovascular artery disease.
- Blood sample for serum lipid profile was collected at the time of admission either in fasting or nonfasting state, and blood sample was sent for biochemical analysis in the department of Biochemistry of RIMS hospital, Ranchi, Jharkhand. The LDL cholesterol was estimated according to the clearance method followed by the enzymatic method (cholesterol oxidase peroxidase method). Total cholesterol, VLDL, HDL cholesterol, and triglycerides were also estimated.
- Patients were diagnosed as clinical FH according to the Dutch Lipid Clinic Network criteria (Table 1) which incorporate clinical history, physical examination, family history, and LDL-C level.

**Table 1:** The Dutch Lipid Clinic Network criteria

Family history	(A) First-degree relative known with premature CAD and/or a first-degree relative with LDL-C > 95th centile	1
	(B) First-degree relative with tendon xanthoma and/or children < 18 with LDL-C > 95th centile	2
Clinical history	(A) Patient has premature CAD	2
	(B) Patient has premature cerebral/peripheral vascular disease	1
Physical examination	Presence of	
	Tendon xanthoma	6
	Arcus cornealis below the age of 45 years	4
LDL-C	(A) > 8.5 mmol/L (more than ~ 330 mg/dl)	8
	(B) 6.5–8.4 mmol/L (~ 250–329 mg/dl)	5
	(C) 5.0–6.4 mmol/L (~ 190–249 mg/dl)	3
	(D) 4.0–4.9 mmol/L(~ 155–189 mg/dl)	1
Definite FH		Score > 8
Probable FH		Score 6–8
Possible FH		Score 3–5
No diagnosis		Score < 3

#### Method of study

The patients with confirmed CAD on coronary angiography were enrolled. Demographic details, clinical history, family history, and treatment history were taken. Physical stigmata of familial hypercholesterolemia like arcus cornealis and tendon xanthoma were examined. Lipid profile was estimated and for a patient on regular cholesterol-lowering medication in whom a pre-treatment LDL-C was not available, and an estimate of untreated LDL-C was obtained by multiplying the measured LDL-C by the validated LDL-C correction factor. Then according to DLCN criteria (Table 1), patients were divided into definite FH, probable FH, possible FH, and no FH groups. This was followed by an analysis of data among the different groups.

#### Data analysis

Descriptive statistics were analyzed with SPSS version 17.0 software. Continuous variables were presented as mean +/− SD. Categorical variables were expressed as frequencies and percentages. Association between two categorical variables was done using Chi-squared or Fisher’s exact test.

## Results

**Fig. 1:**
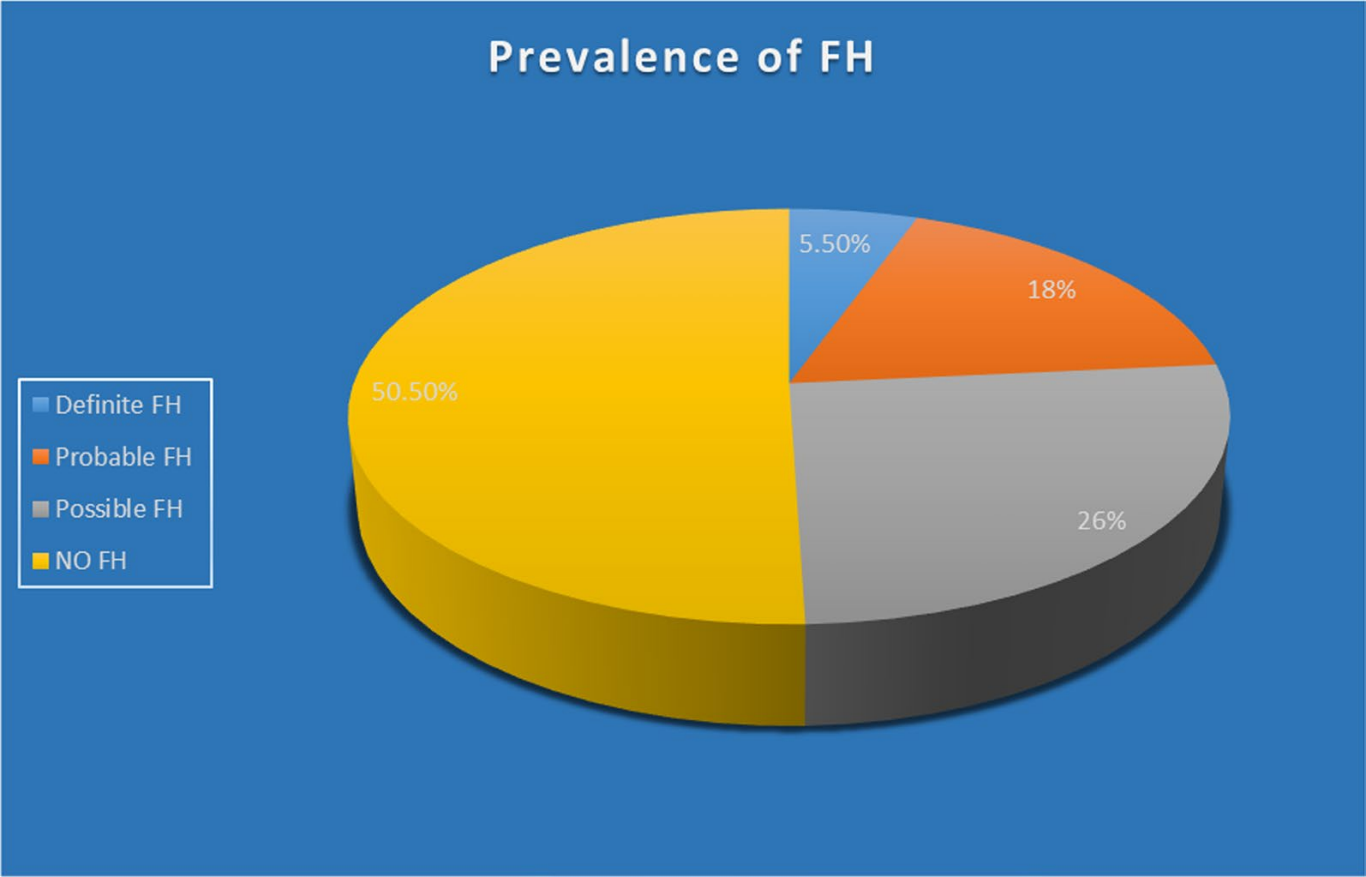
Pie diagram shows the relative percentage of occurrence of different types of familial hypercholesterolemia as classified using the most effective DLCN (Dutch Lipid Clinic Network) criteria because genetic testing was not done by DNA sequencing of candidate genes (LDLR, APOB, PCSK9) in this study

A total of 200 premature CAD patients were included from the OPD/wards/CCU of the Cardiology department of RIMS hospital at Ranchi, Jharkhand. Among the premature CAD patients, 77% were male and the rest were female. In the present study, genetic testing was not carried out by DNA sequencing of candidate genes (LDLR, APOB, PCSK9) and thus premature CAD patients were classified as definite, probable, possible, or no FH (familial hypercholesterolemia) patients only using the most effective DLCN (Dutch Lipid Clinic Network) criteria. The prevalence of different categories present in this study calculated by DLCN criteria points can be summarized in the following pie chart (Fig. 1).

Definite familial hypercholesterolemia in patients with premature CAD was 5.5%, while the prevalence of potential FH [combined definite + probable FH (18%) was 23.5% and the prevalence of possible FH was 26.0% (Table 2).

**Table 2:** Prevalence of potential, possible, and no FH

Types of FH►	Potential FH (Definite + Probable)	Possible FH	No FH
Frequency	47	52	101
Prevalence (%)	23.5	26.0	50.50

The mean age of patients was 40.78 years at the time of enrollment in the hospital, but for patients with definite FH, the same was 35.06 ± 5.64 years, which was younger and statistically significant than other FH categories (Table 3).

**Table 3:** Mean age of FH patients

Types of FH ►	Definite FH	Probable FH	Possible FH	No FH
Number	11	36	52	101
Mean age in years +/− SD	35.0 +/− 5.64	40.41 +/− 4.75	42.51 +/− 7.43	44.39 +/− 5.51

Relatively nonspecific diagnostic sign of AC (Arcus cornealis) was present only in 13% (26) of patients with premature CAD, but among potential FH patients, the incidence of AC was fairly high about 55.31% (26/47) (*p* value < 0.00001) and within definite FH group it was almost 90%.Untreated LDL-C levels from 155 to 189 mg/dL in 18.21% of patients and 190–249 mg/dL in 10% of patients were detected. Coronary angiography showed ~ 36% and 34% of patients having a single (SVD)- and double-vessel disease (DVD), respectively, while TVD was reported in less number of patients (~ 28%). Almost 49% were having a positive family history of premature CAD and 58 (29.0%) patients were smokers. A comorbidity symptom like diabetes was high (42%) as compared to hypertension (28%) in the premature CAD patients. The correlation of these two comorbidities cannot be correlated with the FH-related premature CAD incidence (Tables 4, 5).

**Table 4:** Frequency of diabetes in FH with CAD patients

Category	Definite FH	Probable FH	Possible FH	No FH
*Diabetes*				
YES	2 (18.18%)	7 (19.44%)	24 (46.15%)	52 (51.48%)
NO	9 (81.82%)	29(80.56%)	40 (53.85%)	49 (48.51%)

**Table 5:** Frequency of hypertension in FH patients

Category	Definite FH	Probable FH	Possible FH	No FH
*Hypertension*				
YES	1 (9.09%)	4 (11.11%)	16 (30.76%)	36 (35.64%)
NO	10 (91.0%)	32 (88.89%)	36 (69.23%)	65 (64.36%)

A positive correlation of FH can be drawn with patients having a positive family history of premature CAD (Table 6).

**Table 6:** Frequency of patients having a positive family history of premature CAD

Category	Definite FH	Probable FH	Possible FH	No FH
*Family history of premature CAD*				
YES	10 (90.90%)	32 (88.88%)	45 (86.54%)	0
NO	1 (9.0%)	4 (11.12%)	7 (13.46%)	101 (100%)

Details of all the baseline characteristics of the patients are grouped in Table 7.

**Table 7:** Baseline characteristics

Parameter	Frequency	Percentage (%)
Number of patients	200	
Mean age (in years)	40.78	
Male	154	77.0
Female	46	23.0
*Positive family history of *		
Premature CAD:	97	48.64
Arcus cornealis	26	13.0
Smokers	76	38.0
Hypertension	57	28.50
Diabetes	85	42.50
Past history of CAD:	19	9.50
*Level of LDL-C*		
> 155–189	36	18.21
> 190–249	20	10.0
> 250–329	3	1.5
> 330	3	1.5
LDL correction factor applied:	30	15
*Coronary angiographic finding*		
SVD	73	36.50
DVD	68	34.0
TVD	57	28.50

**Fig. 2:**
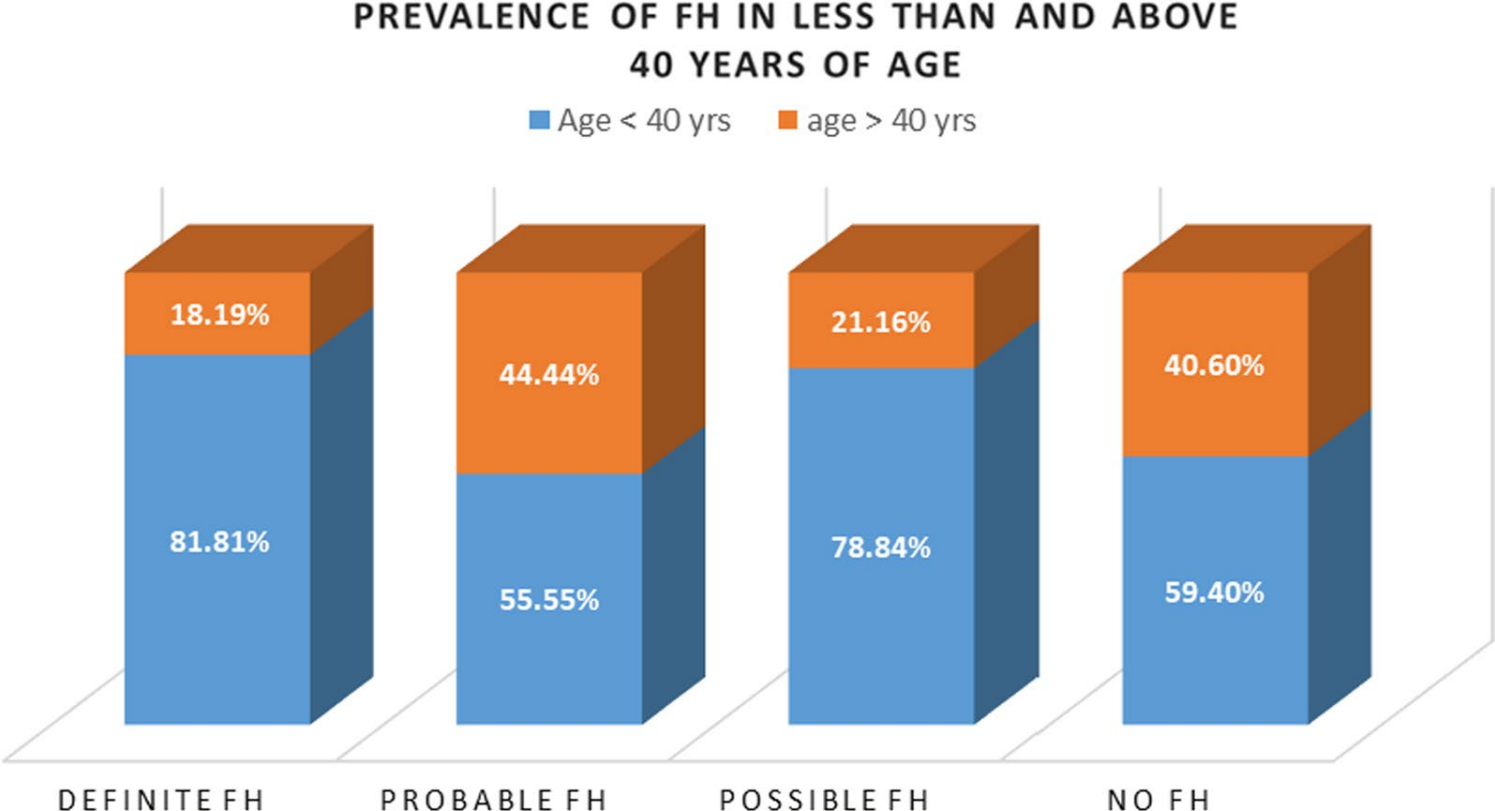
The relative percentage of premature CAD patients that were classified by using the most effective DLCN (Dutch Lipid Clinic Network) criteria grouped into two age categories. The less than 40 years of age group showed more prevalence in definite and possible FH types

The majority of the definite FH group were male patients (10 out of 11), and the comparative age group distribution of different FH groups showed that 81.81% of definite FH belong to the less than 40 years of age cluster (Fig. 2).

**Fig. 3:**
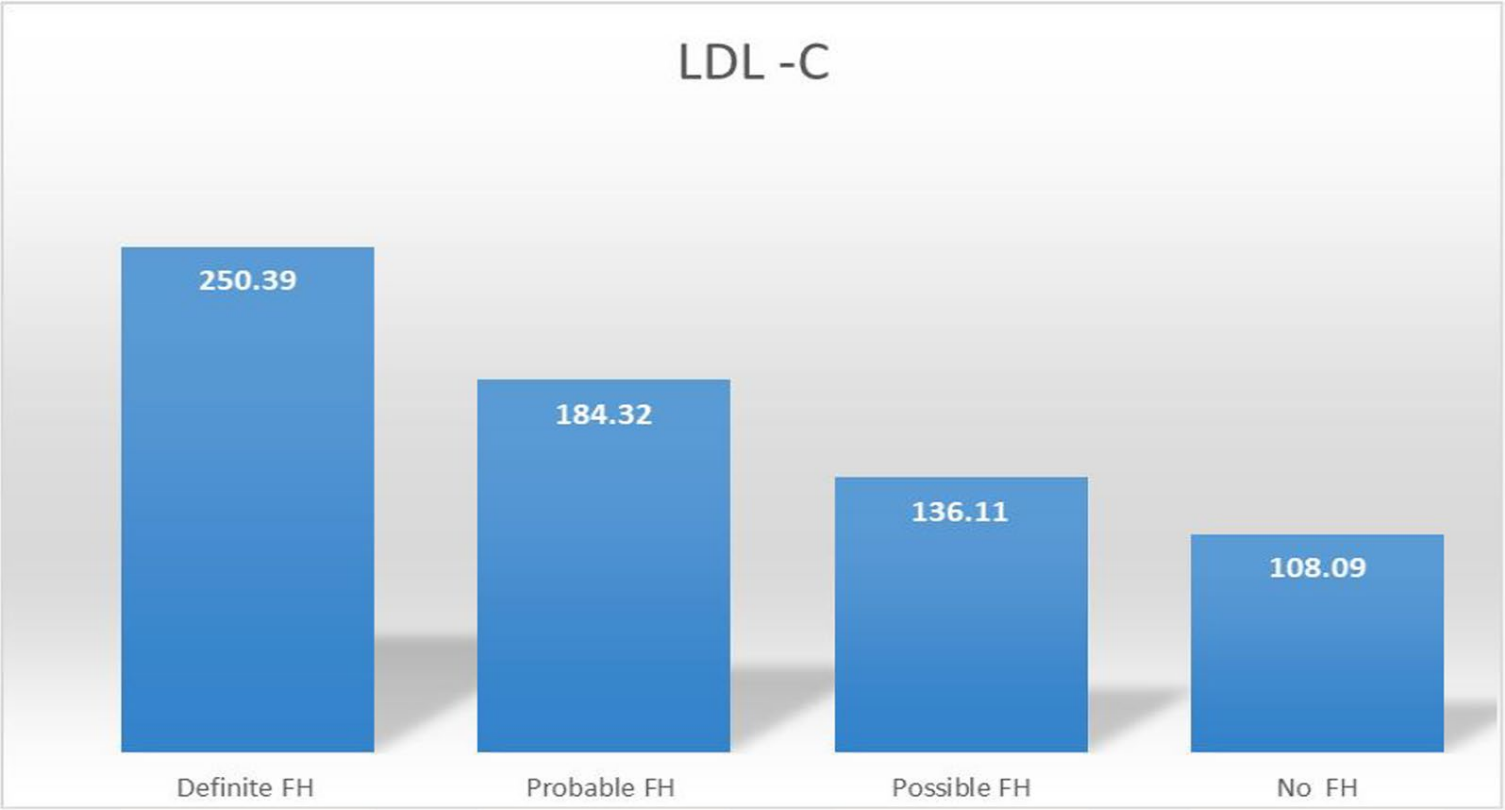
The relation of the presence of mean LDL-C concentration in blood plasma with different types of FH patient groups. The maximum concentration was found in definite FH, and the minimum concentration was found who had no FH detected by DLCN criteria. This finding very well relates the increase in LDL-C directly related to the FH occurrence in premature CAD patients

A statistically significant positive correlation of FH can also be drawn with patients with different levels of LDL-C concentration. Mean concentrations of LDL-C among patients with definite FH, probable FH, possible FH, and no FH group was 250.39 ± 39.88, 184.32 ± 23.41, 136.11 ± 79.72, and 108.09 ± 12.72.72, respectively (Fig. 3).

**Fig. 4:**
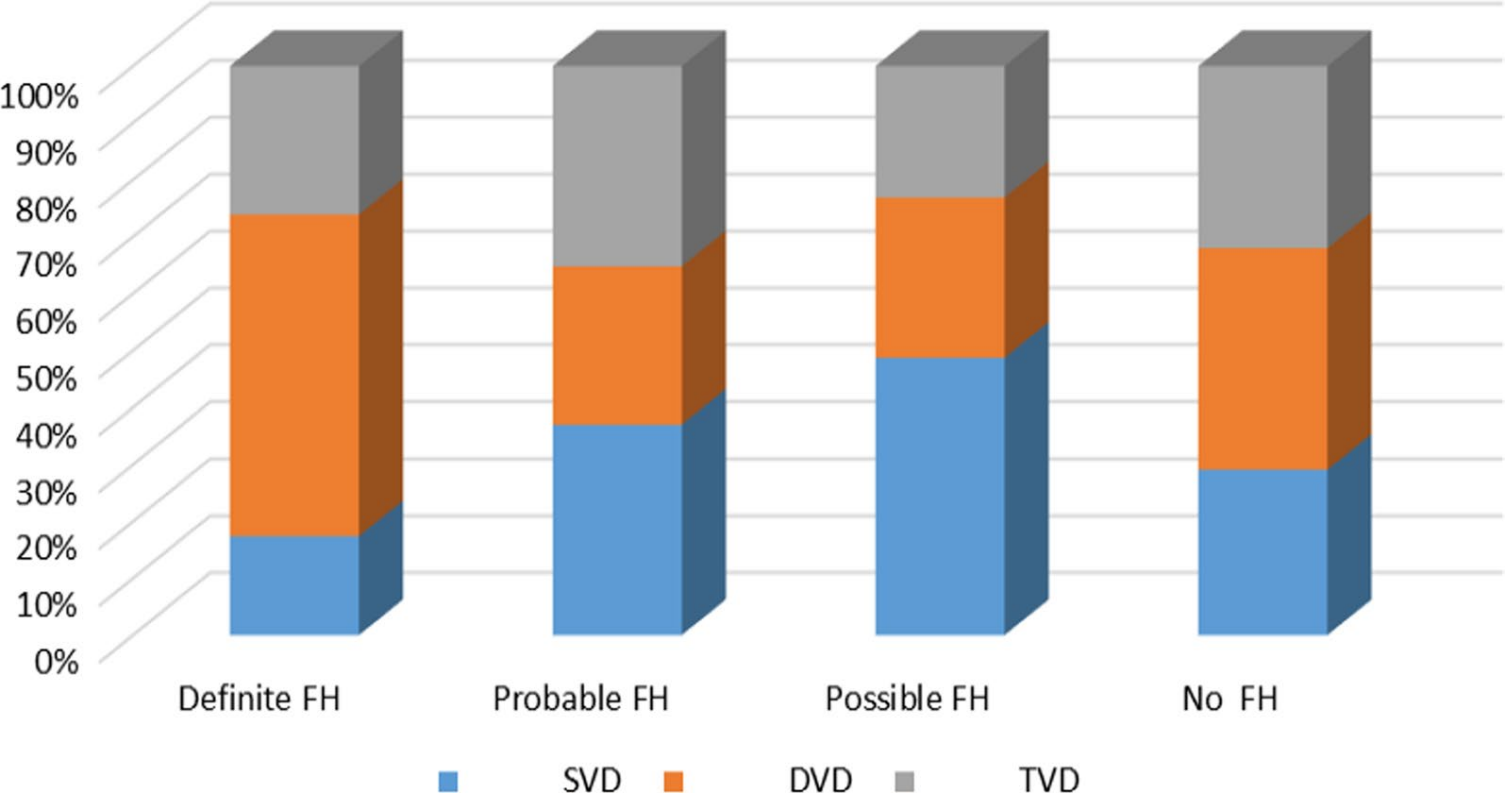
Multi-colored bar diagram showing more occurrences of DVD in definite FH and TVD in probable FH patients, while patients with no FH had an almost equal probability of developing SVD, DVD, and TVD

Coronary angiography findings: Among patients with premature CAD with FH, single-vessel disease (SVD) was more common in probable FH and possible FH groups than in definite FH. Multi-vessel disease (DVD/TVD) was more frequent in the potential FH group (72.34%) than in the possible FH group (51.92%) (*p* < 0.00001) (Fig. 4).

## Discussion

This is the first study to estimate the prevalence of FH in premature coronary artery disease in Jharkhand and may be the first study to address the issue of a large number of patients in India. The diagnosis of FH in patients with premature CAD has been under-recognized throughout India. The present study shows that FH is common in patients with premature coronary artery disease, and it is relatively easily diagnosed clinically without the aid of a genetic screening process.

Out of 200 premature CAD patients attended either in the OPD or wards or CCU of the Department of Cardiology, RIMS, Ranchi, females and males were 46 (23%) and 154 (77.0%) in number, respectively. The mean age of patients was 40.78 ± 6.95 years; the mean age of female and male patients was 47.48 years and 38.64 years, respectively. This ratio showed that the onset age of CAD occurs earlier in male patients than in female patients in Jharkhand. Different types of FH as classified by clinical observations as directed by DLCN criteria were shown to have different percentages of the previous history of CAD in patients in the population of Jharkhand, i.e., 18.18%, 16.70%, 11.30%, and 5.36% patients had a history of the previous CAD in patients diagnosed with definite FH, probable FH, possible FH, and no FH, respectively. It was also observed that 8.5% of the total 200 early CAD patients had a history of dyslipidemia.

The present study showed a high prevalence of FH in premature CAD as compared to previous studies in different parts of the world, based on clinical scoring systems according to DLCN criteria. The prevalence of definite familial hypercholesterolemia was 5.5%, and probable FH was 18.0% in patients with premature coronary artery disease. The prevalence of possible FH was 26.0%, which was fairly high compared to the theoretically estimated percentage of prevalence of heterozygous FH (1/500). The present study showed that the prevalence of potential FH (combining both definite and probable FH) is 23.5% which was even higher than 12.3% as reported by Dorsch et al. [12] among 404 patients of ACS surveyed.

A similar trend of lesser FH was also reported from Singapore by Yudi et al. [13] that included 210 premature CAD patients in the Gold Coast Hospital for 12 months and got 0% definite FH, 1% (3) probable FH, 24% (50) possible FH patients, and 60 (29%) had unlikely FH. The prevalence of FH was very low as compared to the present study. Gaudet et al. [14] reported a genetically confirmed prevalence of 16.4% FH including LPL-C deficient patients among 412 French Canadian men of less than 60 years of age. We report here a prevalence of 23.5% potential FH in premature CAD patients among the population of Ranchi, Jharkhand. Faggiano et al. [15] from Australia studied 175 patients admitted to a coronary care unit with CAD at age < 60 years. Based on modified phenotypic DLCN criteria, the estimated prevalence of probable/definite FH in early onset CAD was 14.3% (95% confidence interval).

In the present study, angiography revealed FH patients with premature CAD exhibited more SVD in the probable (33.33%) and possible (48.07%) FH group than in the definite FH (9%) group. Among multi-vessel diseases, DVD and TVD were more frequent in the potential FH group (23.70% and 31.57%) than in the possible FH group (20.58% and 22.80).

### Limitations

Our present study has several limitations. Firstly, the lipid measurements were performed in different laboratories. An inter-laboratory variation could have conditioned the prevalence of potential FH. Secondly, there were patients in whom the “untreated LDL-C levels” were not measured but estimated using a validated correction formula, and lastly, genetic analysis was not performed in potential FH patients to confirm the diagnosis.

## Conclusions

The present study is the first one to show a high prevalence of FH in premature CAD (about 23.5% for potential FH). To the best of our knowledge, there were no reported large studies of FH or its epidemiology and natural history from Indian patients. Many studies in India have been carried out to study the lipid profile of acute coronary syndrome in Indian patients, but there has been little mention of FH in these publications. Only one previous study, which described clinical profiles and treatment patterns of 997 patients with premature CAD, just briefly mentioned that there was a 1.3% prevalence of possible FH in the study population. This preliminary study revealed that the prevalence of FH in patients with premature CAD who came to the tertiary care hospital of Ranchi, Jharkhand, was high, apart from the conventional risk factors.

## Data Availability

All the raw data and materials mentioned in this manuscript were kept unaffectedly to be produced before the publishing authority, if required.

## Abbreviations

CAD: Coronary artery disease
FH: Familial hypercholesterolemia
DVD: Double-vessel disease
TVD: Triple-vessel disease
SVD: Single-vessel disease
DLCN: Dutch Lipid Clinic Network
ACS: Acute coronary syndrome
OPD: Outpatient department
CCU: Critical care unit
LDL: Low-density lipoprotein
VLDL: Very low-density lipoprotein
HDL: High-density lipoprotein
AC: Arcus cornealis
RIMS: Rajendra Institute of Medical Science
CAG: Coronary artery angiography
HeFH: Heterozygous familial hypercholesterolemia
HoFH: Homozygous familial hypercholesterolemia
APOB: Apolipoprotein B
PCSK9: Proprotein convertase subtilisin/kexin 9
